# Quality of Work Life Factors Among Medical Residents: A Comparative Study of Public and Private Hospitals in Mexico

**DOI:** 10.7759/cureus.115188

**Published:** 2026-08-25

**Authors:** Miguel Angel Martínez Hernandez, Rodolfo Rivas-Ruiz, Lorena Velazquez-Cantor, Guadalupe Graciela Ríos-Flores, Jessica Estefania Silva Guzman

**Affiliations:** 1 Clinical Research, Instituto Mexicano del Seguro Social, Mexico City, MEX; 2 Research and Development, Laboratorios Silanes S.A. de C.V., Mexico City, MEX; 3 Medical, Dental, and Health Sciences Program, Universidad Nacional Autónoma de México, Mexico City, MEX; 4 Medical Education and Research, American British Cowdray Medical Center, Mexico City, MEX

**Keywords:** medical residents, private hospital, public hospital, quality of worklife, workplace violence

## Abstract

Background and objective

Medical residency constitutes a crucial stage in the professional training of physicians, characterized by high clinical and academic demands that directly impact their quality of work life (QoWL). In addition, it is necessary to consider the differences between public and private hospitals, since their organizational structures, availability of resources, and institutional cultures vary significantly, although evidence on how these differences impact QoWL remains limited and inconclusive. The objective of the study was to comprehensively evaluate the factors of the academic-labor environment that affect the QoWL of medical residents in two tertiary-level hospitals in Mexico City, one public and one private.

Materials and methods

A multicenter analytical cross-sectional study was conducted with 325 medical residents from one public and one private hospital in Mexico City. Data collection was carried out through a digital questionnaire including sociodemographic, clinical, and academic-labor variables. Given the self-reported nature of the questionnaires, potential response bias cannot be excluded. To minimize these risks, the survey was conducted digitally and anonymously, and validated instruments such as the brief version of the CVT-GOHISALO and the Inventory of Violence and Psychological Harassment at Work (IVAPt-Pando) were applied to ensure reliability in this population. Statistical analysis included descriptive measures, Pearson’s chi-square (χ²) tests, and multiple logistic regression models.

Results

The overall prevalence of unsatisfactory QoWL was 23.7%, with a higher prevalence in the private hospital (28.8% vs 20.2%). The most affected dimension was free time management (82.5% unsatisfactory). Significant differences were observed in institutional support, with 46.2% of residents in the private hospital reporting dissatisfaction, compared to 19.7% in the public hospital. Logistic regression analyses identified workplace violence as the most critical determinant, increasing more than fourfold the risk of unsatisfactory QoWL. The explained variance of the models rose from 3.8% to 17.4% when organizational and psychosocial factors were included, although other unmeasured factors may also contribute substantially to QoWL.

Conclusions

In conclusion, unsatisfactory QoWL among medical residents in our sample was predominantly associated with psychosocial exposure factors, specifically workplace violence, rather than structural or sociodemographic characteristics. While our observational design limits normative recommendations, these results highlight workplace violence and institutional support as key targets for future longitudinal research and institutional policy evaluation.

## Introduction

Medical residency constitutes a crucial stage in the professional training of physicians, characterized by the dual condition of both an academic specialization process and, at the same time, a formal employment relationship with hospital institutions. This period is distinguished by high clinical and academic demands, which make resident physicians “special workers”, subjected to a dynamic and complex hospital environment that directly impacts their well-being and their quality of work life (QoWL) [1,2].

This academic-labor environment of residents is characterized by constant exposure to psychosocial, biological, chemical, ergonomic, and physical risks, in addition to high work demands, lack of institutional support, vertical and horizontal violence, exposure to pathogens and chemical substances, as well as physical overload derived from repetitive and prolonged tasks. These factors, together with the scarcity of material and human resources, role ambiguity, and lack of motivational incentives, generate tensions that affect the physical and mental health of residents, impacting their learning capacity and professional performance [3,4].

Likewise, the context outside the hospital cannot be ignored, since aspects such as social relationships, free time, hobbies, and the community environment significantly influence the perception of well-being and QoWL. The constant contact, of more than 80 hours of activity per week, with suffering, uncertainty, and the responsibility of medical care intensifies the emotional burden, turning residency into a period of psychological and physical vulnerability [5].

In addition, it is necessary to consider the differences between public and private hospitals, since their organizational structures, availability of resources, and institutional cultures vary significantly. Although it is commonly assumed that private hospitals offer better conditions for medical residents, the available evidence on how these differences impact QoWL is limited and inconclusive. Most studies have focused on describing workload and psychosocial factors in general, without delving into the particularities that distinguish the private sector from the public [6,7].

In this sense, the objective of the study was to comprehensively evaluate the factors of the academic-labor environment that affect the QoWL of medical residents in two tertiary-level hospitals in Mexico City, one public and one private. We hypothesized that, despite structural and material differences between hospital sectors, organizational exposures and psychosocial dynamics, specifically workplace violence, exert a greater influence on residents' QoWL than institutional ownership per se.

## Materials and methods

Study design and data sources

A multicenter analytical cross-sectional study was conducted, which was submitted for review and approval by the corresponding Ethics Committees, thereby ensuring compliance with national and institutional ethical guidelines. Initially, it was registered with the National Scientific Research Committee of the Mexican Social Security Institute (approval number R-2022-785-020) and with the Research and Research Ethics Committee of the American British Cowdray Medical Center (approval number CMABC-23-01), both located in Mexico City.

The study was conducted at two tertiary-level teaching hospitals in Mexico City: a public tertiary center (a 158-bed unit of medical high specialty providing highly complex care to public healthcare beneficiaries) and a private tertiary center (over 400 beds across its campuses, offering comprehensive highly specialized care for private-insurance and out-of-pocket patients). Both institutions maintain formal medical residency programs accredited by the National Autonomous University of Mexico.

We included active medical residents enrolled in accredited training programs at either institution who provided informed consent during the data collection period. Residents were excluded if they were on administrative leave, sick leave, or external rotations away from the main hospital facilities during the study. Participants were recruited through an open invitation and voluntary participation strategy. The invitation to participate was extended during in-person academic sessions and through electronic means, ensuring broad and equitable outreach. Each candidate was provided with detailed information regarding the protection of personal data and, upon agreeing to participate, proceeded to sign a digital informed consent form. Subsequently, participants completed a cross-sectional survey aimed at identifying the initial level of QoWL and the exposure factors present in their respective hospital centers.

Definition of variables

The data collection process was carried out through a digital questionnaire, designed to facilitate self-administration on any electronic device (Appendix A). The first page of the form included a statement on the protection of personal data, followed by a comprehensive clinical and academic-labor history. The primary outcome was unsatisfactory QoWL, evaluated both globally and across seven specific dimensions using the validated CVT-GOHISALO scale. This instrument evaluates seven dimensions of QoWL: institutional support, job security, job integration, job satisfaction, work-related well-being, personal development, and free time management. Each dimension was measured through items with a Likert-type scale, which allowed for standardized and comparative assessment [8].

Complementarily, the Inventory of Violence and Psychological Harassment at Work (IVAPT-Pando), composed of 22 items and validated for the Mexican population, was applied [9]. Scores were originally interpreted according to the author’s validated risk cutoffs (no exposure/low, moderate, and high/severe violence). Workplace violence exposure was operationalized as a binary outcome (presence vs. absence), where scores reaching or exceeding the author’s threshold for moderate-to-severe workplace violence were classified as presence of exposure. 

The average response time for the questionnaires was 18 to 20 minutes. Upon completion, participants automatically received in their email a copy of their responses and a permanent link to consult the informed consent, reinforcing transparency and continuous access to information.

The variables collected included sociodemographic data such as sex and age; clinical characteristics, including physical comorbidities and mental health history; and academic-labor aspects, such as length of service in the hospital, medical specialty, work-related disabilities in the last year, and perception of risk factors in the work environment. These factors encompassed physical, chemical, biological, ergonomic, and psychosocial risks. The methodological decision to evaluate subjective perception was based on Karasek’s demand-control model, which recognizes perception as a marker of the effect of physical and psychosocial exposure at work [10].

Likewise, additional ethical measures were established for the protection of participants. In cases where a severe mental health diagnosis was identified, they were referred to the psychology service of the Occupational Medicine Department, according to their institution. This ensured timely and appropriate complementary management, in accordance with the principles of beneficence and justice that govern health research.

Statistical analysis

To calculate the required sample size, the Lwanga-Lemeshow formula was used for the comparison of proportions of independent populations, considering a 95% confidence level, 80% statistical power, and a minimum expected difference of 15% between the public and private sectors.

As an open invitation model was used, the final sample represents a voluntary, convenience-based dataset of all residents who actively responded to the call. All submitted questionnaires were verified for completeness upon collection, resulting in a complete-case dataset with no missing items.

An initial descriptive analysis was performed to determine the clinical, demographic, and labor characteristics of the medical residents. The distribution of the variables age and length of service was analyzed by evaluating skewness and kurtosis and confirmed through the Kolmogorov-Smirnov normality test. Both variables present a free distribution; therefore, they are presented as median (p50) and percentiles (p25 and p75). In addition, qualitative variables are shown as frequencies and proportions.

A homogeneity analysis between both hospital centers was carried out. For this purpose, the Mann-Whitney U test was used for the variables age and length of service, and Pearson’s chi-square (χ^2^) test (or Fisher’s exact test in cases where any cell had an expected count less than 5). Heterogeneity between groups was considered for those where the p-value was less than 0.05. The overall QoWL level and its dimensions were compared between both hospitals using Pearson’s χ^2^ test.

Several models were carried out through multiple binary logistic regression analysis using the “enter” method, where p-values, measures of association, and 95% CIs were analyzed.

To preserve the stability of the model, dummy variables were generated from the different factors to which medical residents perceived themselves to be exposed: a) Physical factors for noise, vibration from a device, poor lighting, extreme temperatures (low or high), and radiation from an artifact; b) Chemical factors for dust, electrosurgical smoke, anesthetic gases or vapors, and liquids such as solvents or acids; c) Ergonomic factors for forced postures, repetitive movements, poor distribution of workspace, prolonged work standing, sitting, or in other positions. Each factor was dichotomized according to its absence or presence.

The nature of each variable was considered for this modeling. In the first model, individual factors were included. In the second model, Model 1 was adjusted for clinical factors. The third model included Model 2 and labor structure factors such as hospital center, medical service branch, and length of service. In the fourth model, Model 3 was adjusted for perceived exposure given by the workplace; and finally, in a fifth model, Model 4 was adjusted for the psychosocial work environment (workplace violence). For all models, Nagelkerke’s R² and the Hosmer-Lemeshow goodness-of-fit test were analyzed.

## Results

A sample of 325 medical residents from two hospital centers was analyzed, with a median age of 29 years and a prevalence of 56.6% men (n=184). When comparing both institutions, statistically significant differences were observed in sex, with a higher prevalence of women in the private center compared to the public center.

Likewise, differences were observed in baseline health-disease states; medical residents from the public hospital reported a higher frequency of physical comorbidity diagnoses (n=76, 39.4% vs. n=17, 12.9%), while in the private hospital a higher prevalence of history of mental health disorders was found (n=46, 34.8% vs. n=25, 13%).

In addition, greater heterogeneity was observed among the branches of medical service within the private hospital. Regarding the hospital environment, the private hospital showed a higher perception of exposure to occupational risk factors, including noise (n=71, 53.8%), extreme temperatures (n=60, 45.5%), workplace violence (n=62, 47%), and forced postures (n=61, 46.2%), with statistically significant differences compared to the public hospital (Table 1).

**Table 1:** Individual, clinical, and labor characterization of medical residents from both hospital centers ^1^p50 (p25, p75);^ 2^n (%); ^a^Mann-Whitney U test;^ b^Pearson's chi-square (χ^2^) test; ^c^Fisher's exact test; *Statistically significant (p<0.05).

Characteristics		Total (n=325)	Public hospital center (n=193)	Private hospital center (n=132)	Test statistics	p-value
Age ^1^		29 (28-31)	29 (28-31)	29 (28-31)	13161 ^a^	0.607
Sex ^2^	Male	184 (56.6)	121 (62.7)	63 (47.7)	7.15 ^b^	0.008 *
	Female	141 (43.4)	72 (37.3)	69 (52.3)		
History of mental health disorders ^2^	Without history	254 (78.2)	168 (87)	86 (65.2)	22.010 ^b^	<0.001 *
	With history	71 (21.8)	25 (13)	46 (34.8)		
Diagnosed physical comorbidities ^2^	Without history	232 (71.4)	117 (60.6)	115 (87.1)	26.95 ^b^	<0.001 *
	With history	93 (28.6)	76 (39.4)	17 (12.9)		
Medical service branch ^2^	Paraclinical	33 (10.2)	17 (8.8)	16 (12.1)	94.70 ^b^	<0.001 ^b^ *
	Clinical	193 (59.4)	154 (79.8)	39 (29.5)		
	Surgical	53 (16.3)	7 (3.6)	46 (34.8)		
	Critical care	46 (14.2)	15 (7.8)	31 (23.5)		
Job seniority at the hospital ^1^		2 (1-3)	2 (1-3)	2 (1-3)	13759.5 ^a^	0.206
Leave due to general illness in the last year ^2^		8 (2.5)	0 (0)	8 (6.1)	N/A ^c^	<0.001 *
Leave due to occupational illness in the last year ^2^		6 (1.8)	6 (3.1)	0 (0)	N/A ^c^	<0.001 *
Perception of work-exposure to: ^2 ^	Noise	117 (36)	46 (23.8)	71 (53.8)	30.53	<0.001 ^b^ *
	Vibrations	55 (16.9)	21 (10.9)	34 (25.8)	12.34 ^b^	<0.001 *
	Poor lighting ^2^	67 (20.6)	31 (16.1)	36 (27.3)	6.02 ^b^	0.014 *
	Extreme temperatures ^2^	81 (24.9)	21 (10.9)	60 (45.5)	50.08 ^b^	<0.001 *
	Radiation	141 (43.4)	79 (40.9)	62 (47)	1.16 ^b^	0.281
	Dusts from medication preparation	50 (15.4)	29 (15)	21 (15.9)	0.047 ^b^	0.828
	Electrosurgical fumes	53 (16.3)	13 (6.7)	40 (30.3)	31.90 ^b^	<0.001 *
	Anesthetic gases or vapors	48 (14.8)	23 (11.9)	25 (18.9)	3.07 ^b^	0.080
	Liquids such as solvents or acids	39 (12)	30 (15.5)	9 (6.8)	5.65 ^b^	0.017 *
	Biologicals such as viruses, bacteria, or fungi	204 (62.8)	122 (63.2)	82 (62.1)	0.04 ^b^	0.842
	Poor workspace distribution	94 (28.9)	39 (20.2)	55 (41.7)	17.56 ^b^	<0.001 *
	Forced postures	130 (40)	69 (35.8)	61 (46.2)	3.58 ^b^	0.059
	Repetitive movements	87 (26.8)	27 (14)	60 (45.5)	39.59 ^b^	<0.001 *
	Prolonged standing work	200 (61.5)	109 (56.5)	91 (68.9)	5.14 ^b^	0.023 *
	Prolonged sitting work	137 (42.2)	64 (33.2)	73 (55.3)	15.76 ^b^	<0.001 *
	Prolonged work in other positions	44 (13.5)	9 (4.7)	35 (26.5%)	31.98 ^b^	<0.001 *
	Workplace violence (harassment, bullying, or mistreatment)	81 (25)	19 (9.9)	62 (47)	57.34 ^b^	<0.001 *

The overall prevalence of unsatisfactory QoWL was 23.7% (n=77), with a higher prevalence in the private hospital (n=38, 28.8% vs. n=39, 20.2%). The most affected dimension was free time management, where 82.5% (n=268) of residents reported unsatisfactory results. Statistically significant differences were found in the dimension of institutional support, where 46.2% (n=61) of medical residents in the private hospital perceived unsatisfactory support, compared to only 19.7% (n=38) in the public hospital (Table 2).

**Table 2:** Comparison of the level of overall quality of work life (QoWL) and by dimensions between the two hospitals Data are presented as n (%); ^a^Pearson’s chi-square (χ^2^) test; *Statistically significant (p<0.05).

Dimensions of QoWL		Total (n=325)	Public hospital center (n=193)	Private hospital center (n=132)	Test statistics ^a^	p-value
QoWL (global)	Satisfactory	248 (76.3)	154 (79.8)	94 (71.2)	3.192	0.074
	Non-satisfactory	77 (23.7)	39 (20.2)	38 (28.8)		
Institutional support for work	Satisfactory	226 (69.5)	155 (80.3)	71 (53.8)	26.033	<0.001 *
	Non-satisfactory	99 (30.5)	38 (19.7)	61 (46.2)		
Safety at work	Satisfactory	198 (60.9)	118 (61.1)	80 (60.6)	0.009	0.923
	Non-satisfactory	127 (39.1)	75 (38.9)	52 (39.4)		
Integration into the workplace	Satisfactory	252 (77.5)	154 (79.8)	98 (74.2)	1.387	0.239
	Non-satisfactory	73 (22.5)	39 (20.2)	34 (25.8)		
Job satisfaction	Satisfactory	261 (80.3)	160 (82.9)	101 (76.5)	2.022	0.155
	Non-satisfactory	64 (19.7)	33 (17.1)	31 (23.5)		
Well-being achieved through work	Satisfactory	285 (87.7)	164 (85)	121 (91.7)	3.253	0.071
	Non-satisfactory	40 (12.3)	29 (15)	11 (8.3)		
Personal development of the worker	Satisfactory	242 (74.5)	152 (78.8)	90 (68.2)	4.610	0.032 *
	Non-satisfactory	83 (25.5)	41 (21.2)	42 (31.8)		
Free time management	Satisfactory	57 (17.5)	35 (18.1)	22 (16.7)	0.117	0.733
	Non-satisfactory	268 (82.5)	158 (81.9)	110 (83.3)		

Different logistic regression analyses were carried out according to the nature of the variables. In the first three models, individual variables, clinical history, and labor structure characteristics were included, where no statistically significant associations were found.

However, when including in the analysis the perceptions of exposure to occupational risk factors, as well as the psychosocial environment, certain findings were observed. From Model 4 onward, the presence of physical comorbidities showed an association with the outcome (OR=0.488, 95% CI 0.246-0.969), which persisted in the final model (OR=0.475, 95% CI 0.236-0.957). In Model 4, the perception of exposure to ergonomic factors was associated with unsatisfactory QoWL (OR=2.403, 95% CI 1.128-5.122); however, this statistical significance was lost in Model 5 when including the variable of workplace violence. This latter variable represents a 4.17-fold higher risk of presenting unsatisfactory QoWL compared to those who do not perceive it (OR=4.174, 95% CI 2.091-8.333).

The explained variance of the model increased when including workplace conditions, rising from 3.8% in Model 3 to 17.4% in Model 5. All models showed adequate goodness of fit, according to the Hosmer-Lemeshow test (Table 3).

**Table 3:** Evaluation of the main factors associated with unsatisfactory quality of work life Model 1 (Individual factors): Nagelkerke R² = 0.014, Hosmer-Lemeshow p = 0.377; Model 2 (Model 1 + clinical factors): Nagelkerke R² = 0.031, Hosmer-Lemeshow p = 0.999; Model 3 (Model 2 + labor structure): Nagelkerke R² = 0.038, Hosmer-Lemeshow p = 0.649; Model 4 (Model 3 + workplace exposure): Nagelkerke R² = 0.101, Hosmer-Lemeshow p = 0.455; Model 5 (Model 4 + psychosocial work environment): Nagelkerke R² = 0.174, Hosmer-Lemeshow p = 0.363

Characteristics		Model 1	Model 2	Model 3	Model 4	Model 5
Age		0.920 (0.828-1.022)	0.923 (0.831-1.026)	0.919 (0.823-1.026)	0.934 (0.837-1.043)	0.938 (0.838-1.050)
Sex	Male	Ref.	Ref.	Ref.	Ref.	Ref.
	Female	1.202 (0.718-2.013)	1.204 (0.713-2.031)	1.145 (0.672-1.950)	1.029 (0.594-1.785)	0.995 (0.562-1.764)
History of mental health disorders	Without history		Ref.	Ref.	Ref.	Ref.
	With history		1.215 (0.655-2.256)	1.076 (0.558-2.074)	1.013 (0.519-1.977)	0.795 (0.394-1.606)
Diagnosed physical comorbidities	Without history		Ref.	Ref.	Ref.	Ref.
	With history		0.566 (0.305-1.050)	0.620 (0.320-1.201)	0.488 (0.246-0.969)	0.475 (0.236-0.957)
Hospital center	Public			Ref.	Ref.	Ref.
	Private			1.357 (0.694-2.653)	1.049 (0.517-2.130)	0.643 (0.296-1.397)
Medical service branch	Paraclinical			Ref.	Ref.	Ref.
	Clinical			0.911 (0.371-2.241)	0.913 (0.355-2.349)	0.687 (0.262-1.802)
	Surgical			0.877 (0.308-2.499)	0.613 (0.204-1.846)	0.447 (0.140-1.427)
	Critical care			1.152 (0.406-3.272)	1.118 (0.383-3.267)	0.729 (0.238-2.232)
Job seniority at the hospital				0.986 (0.827-1.177)	0.969 (0.805-1.166)	0.945 (0.778-1.148)
Exposure to physical factors	No exposure				Ref.	Ref.
	Exposure to ≥1 factor				1.828 (0.920-3.633)	1.694 (0.828-3.468)
Exposure to chemical factors	No exposure				Ref.	Ref.
	Exposure to ≥1 factor				1.354 (0.736-2.491)	1.403 (0.742-2.653)
Exposure to biological factors	No exposure				Ref.	Ref.
	Exposure to ≥1 factor				0.966 (0.539-1.729)	0.869 (0.476-1.586)
Exposure to ergonomic factors	No exposure				Ref.	Ref.
	Exposure to ≥1 factor				2.403 (1.128-5.122)	2.051 (0.945-4.455)
Workplace violence exposure	No exposure					Ref.
	With exposure					4.175 (2.091-8.333)

## Discussion

The present study analyzed a sample of 325 medical residents from two hospital centers, revealing an overall unsatisfactory QoWL of 23.7% (n=77), with 20.2% (n=39) in the public hospital and 28.8% (n=38) in the private hospital. Although the overall percentage could be interpreted as a majority positive perception, the stratified analysis by dimensions and by institution exposed significant vulnerabilities that warrant discussion. This finding confirms that individual characteristics, such as age, sex, and the basic conditions of labor structure, are not sufficient to understand unsatisfactory QoWL and reinforces the idea that it is a construct defined primarily by human intention and organizational culture, rather than by individual attributes of the resident. In other words, QoWL reflects a complex interplay of workplace and psychosocial conditions, where interpersonal interactions, institutional support structures, and the daily work environment of the physician in training play a central role [11].

When comparing both institutions, statistically significant differences were observed in sex distribution, with a higher proportion of women in the private hospital. The observed baseline disparities between institutions reflect structural differences in residency program composition and specialty distribution across healthcare sectors in Mexico. This finding is relevant because it introduces a gender component in the interpretation of QoWL, given that the literature has indicated that female residents face greater challenges in terms of work-life balance, as well as in exposure to workplace violence dynamics [12]. 

Likewise, differences were identified in baseline health-disease states: residents of the public hospital reported a higher frequency of physical comorbidities (n=76, 39.4% vs. n=17, 12.9%), while in the private hospital a higher prevalence of a history of mental health disorders was observed (n=46, 34.8% vs. n=25, 13%). This divergence presents an interesting contrast: in the public hospital, risks appear to be more linked to previous physical conditions, whereas in the private hospital, residents face a greater burden of psychosocial factors. This difference could be related to the characteristics of the work environment, since the private hospital showed a higher perception of exposure to risk factors such as noise (n=71, 53.8%), extreme temperatures (n=60, 45.5%), workplace violence (n=62, 47%), and forced postures (n=61, 46.2%) [13].

The most affected dimension of QoWL was free time management, where 82.5% of residents reported unsatisfactory results. This finding confirms that work overload and the lack of recovery spaces are major problems in medical training. However, the most significant difference was observed in the dimension of institutional support: 46.2% of residents in the private hospital perceived unsatisfactory support, compared to 19.7% in the public hospital. This result highlights a notable contrast: despite the private sector often offering superior physical infrastructure, residents in this setting reported lower perceived institutional support. While the underlying organizational mechanisms warrant further investigation, this disparity suggests that material conditions alone do not guarantee a supportive training environment, directly impacting residents' overall perception of QoWL [14].

Logistic regression analyses provided additional evidence on the factors associated with unsatisfactory QoWL. In the first models, which included individual variables, clinical history, and labor structure characteristics, no significant associations were found. Notably, although sex showed a baseline imbalance between hospital sectors, it did not demonstrate a statistically significant independent association with unsatisfactory QoWL across any of the multivariable models. When incorporating perceptions of exposure to occupational risk factors and the psychosocial environment, key findings emerged. The presence of physical comorbidities showed an inverse association with unsatisfactory QoWL (OR = 0.475, 95% CI 0.236-0.957), suggesting that residents with physical illnesses may receive some degree of institutional support or develop coping strategies that buffer the impact on their QoWL [14].

On the other hand, the perception of exposure to ergonomic factors was initially associated with unsatisfactory QoWL (OR = 2.403, 95% CI 1.128-5.122), although this significance was lost when including the variable of workplace violence in the final model. Therefore, workplace violence was consolidated as the most critical factor, increasing more than fourfold the risk of presenting unsatisfactory QoWL (OR = 4.174, 95% CI 2.091-8.333). This finding confirms that, although the physical conditions of the environment are relevant, systematic mistreatment and hostile environments have a more devastating impact on the perception of labor well-being [15].

In the same way, the explained variance of the model increased notably when including workplace conditions, rising from 3.8% in Model 3 to 17.4% in Model 5. Although a Nagelkerke R² of 17.4% reflects a modest overall proportion of explained variance, which indicates that QoWL is a multi-dimensional construct influenced by additional unmeasured personal and environmental factors, this increase shows that QoWL is a construct defined mainly by organizational and psychosocial factors rather than by individual characteristics. All models showed adequate goodness of fit, which reinforces the robustness of the findings.

Crucially, in our multivariable regression modeling, the crude association between hospital type and unsatisfactory QoWL lost statistical significance once organizational exposures, sex, and psychosocial variables were introduced (Model 5, OR = 0.643, 95% CI: 0.296-1.397). This attenuation demonstrates that neither hospital ownership per se nor baseline demographic imbalances, including sex distribution, are independent risk factors for poor QoWL. Rather, after adjusting for sex and clinical history, crude inter-hospital differences are largely mediated by the striking disparity in perceived workplace violence.

Taken together, the results of this study indicate that the QoWL of medical residents is associated with a range of clinical, physical, and psychosocial parameters, with workplace violence emerging as the primary independent factor [11,15]. Furthermore, the observed differences between sectors suggest that favorable physical infrastructure does not automatically correspond to high perceived institutional support, highlighting the importance of addressing interpersonal dynamics and workplace environment alongside material resources [7].

Based on our findings, targeted institutional strategies addressing the primary factors identified in our models, particularly workplace violence prevention and ergonomic risk mitigation, may play a crucial role in improving residents' QoWL. Programs of psychosocial support, such as collective exchange spaces, are valuable to mitigate the perception of vulnerability. While broader organizational interventions were not directly evaluated in this study, our data suggest that strengthening institutional support systems is an essential component to consider alongside physical workplace improvements [16].

Among the limitations of our study are those inherent to the cross-sectional design, which restricts the establishment of temporal relationships between variables. To address this limitation, time modeling was carried out, with a theoretical reconstruction of exposure according to the nature of the variables themselves. Likewise, voluntary participation and the invitation through academic sessions and electronic media may have generated selection bias, favoring the inclusion of residents with greater interest in the subject or particular experiences. In addition, the reliance on self-reported questionnaires may have introduced bias. To mitigate this issue, data were collected anonymously through a digital platform to foster honest reporting and eliminate interviewer influence. Furthermore, measurement relied on validated scales psychometrically tested for healthcare professionals, which helps preserve structural validity despite the subjective nature of self-reported outcomes. Therefore, we suggest that future studies use probabilistic sampling strategies and consider complementary data collection methods to further reduce these biases.

Finally, the potential for residual confounding must be acknowledged. Due to institutional administrative policies that limited data collection for specific variables at one of the participating centers, key parameters such as workload intensity, frequency and duration of on-call shifts, income or compensation differences between institutions, sleep quality, individual coping strategies, and external psychosocial support systems were not directly evaluated in our models. Because these unmeasured variables are key drivers of medical resident well-being, future prospective studies should integrate them alongside institutional parameters to capture the full spectrum of QoWL.

## Conclusions

The QoWL of medical residents results from a multicausal interaction in which psychosocial factors, such as workplace violence, have a determining weight over physical and ergonomic factors, consolidating as the main element that deteriorates well-being during medical training. The institutional paradox demonstrates that better material conditions and administrative efficiency do not replace organizational support, confirming that infrastructure, occupational exposure, and institutional culture define the experience of the medical resident. In conclusion, unsatisfactory QoWL among medical residents in our sample was predominantly associated with psychosocial exposure factors, specifically workplace violence, rather than structural or sociodemographic characteristics. While our observational design limits normative recommendations, these results highlight workplace violence and institutional support as key targets for future longitudinal research and institutional policy evaluation.

## Appendices

Appendix A

Study Questionnnaire

The data collection process was carried out through a digital questionnaire, designed to facilitate self-administration on any electronic device.

Section 1: Personal & Demographic Information

What is your age? *(completed years) *______________________________________

What is your sex?

Select one

◯ Male

◯ Female

What is your current marital status?

Select one

◯ Single / Without a partner

◯ In a relationship / With a partner

◯ Married

How often do you play a sport or exercise?

Select one

◯ Daily

◯ 2 to 3 times a week

◯ Occasionally or never

Your daily physical activity, both at the hospital and in daily life activities, is:

Select one

◯ Sedentary

◯ Light

◯ Moderate

◯ Intense

◯ Extremely intense

Do you smoke?

Select one

◯ No, I have never smoked

◯ Yes, occasionally

◯ Yes, I smoke daily

Do you consume alcohol?

Select one

◯ No, I never drink

◯ Yes, occasionally

◯ Yes, frequently (at least once a month)

In your free time, do you consume or use any other type of psychoactive substance?

Select one

◯ Never

◯ Occasionally

◯ Frequently

How do you consider your eating habits in terms of energy balance and schedule?

Select one

◯ Good

◯ Fair

◯ Poor

Section 2: Health Status & Conditions

Do you have a medical diagnosis for any of the following conditions?

◯ Attention-Deficit/Hyperactivity Disorder (ADHD)

◯ Major Depressive Disorder

◯ Generalized Anxiety Disorder

◯ Sleep disorder

◯ Chronic illness (Diabetes, hypertension, thyroid pathology, cancer)

If you selected "Other", please specify: ______________________________________

Section 3: Work Background

Service area where you are currently working within the hospital: 

◯ Anatomic Pathology

◯ Anesthesiology

◯ Bariatric Surgery

◯ Breast Imaging and Intervention

◯ Cardiology

◯ Cerebrovascular Disease

◯ Clinical Neurophysiology

◯ Computed Tomography

◯ Critical Care Medicine

◯ Diagnostic and Therapeutic Radiology

◯ Emergency Medicine

◯ Endoscopic Surgery

◯ Foot and Ankle Surgery

◯ General Surgery

◯ Geriatrics

◯ Hematopoietic Progenitor Cell Transplantation

◯ Immunohistochemistry in Surgical Pathology

◯ Internal Medicine

◯ Laparoscopic and Robotic Surgery in Urology

◯ Magnetic Resonance Imaging

◯ Medical Oncology

◯ Nuclear Medicine and Molecular Imaging

◯ Obstetrics and Gynecology

◯ Pain and Palliative Medicine

◯ Pediatrics

◯ Perioperative Medicine in Obese Patients

◯ Positron Emission Tomography (PET-CT)

◯ Spine Surgery

◯ Therapeutic Gastrointestinal Endoscopy

◯ Traumatology and Orthopedics

◯ Urology

Which year of residency are you currently completing?

Select one

◯ PGY-1 

◯ PGY-2 

◯ PGY-3

◯ PGY-4 

◯ PGY-5 or higher

In the last year:

◯ Have you taken sick leave due to a general illness?

◯ Have you taken sick leave due to an illness caused by your work at the hospital?

◯ Have you taken sick leave due to an accident that occurred during your work at the hospital?

In your current job position, are you frequently exposed to high levels of:

(Please select all options that apply)

◯ Noise

◯ Vibration from any device/equipment

◯ Poor lighting

◯ Very high temperatures

◯ Very low temperatures

◯ Radiation from any device

◯ Dust

◯ Electrosurgical smoke

◯ Anesthetic gases or vapors

◯ Liquids such as solvents or acids

◯ Biological hazards such as viruses, bacteria...

◯ Mistreatment or any type of violence [from superiors]

◯ Mistreatment or any type of violence [from coworkers]

◯ Mistreatment or any type of violence [from subordinates]

◯ Mistreatment or any type of violence [from patients/relatives]

◯ Forced postures

◯ Physical overexertion

◯ Repetitive movements

◯ Poor workspace distribution

◯ Prolonged standing work

◯ Prolonged sitting work

◯ Prolonged work in other body positions

Section 4: Quality of Work Life (CVT-GOHILSALO)

Mark the response that best describes your situation. The following questions reflect your degree of satisfaction regarding the indicated topics:

Response Options (Likert Scale)

Not satisfied at all

Slightly satisfied

Moderately satisfied

Satisfied

Very satisfied

--

Statements:

This is the level of satisfaction I have regarding the process followed to supervise my work

It is the degree of satisfaction I have regarding the treatment I receive from my superiors

I have been clearly and precisely instructed on how to do my job

I receive feedback from my colleagues and superiors regarding their evaluation of my work

In my institution, efficiency and preparation efforts are recognized with promotion opportunities

I consider that I have the freedom to express my opinions regarding work without fear of retaliation from my bosses

It is the degree of satisfaction I feel with the way procedures to perform my work are designed

My level of satisfaction regarding my current salary is

It is my level of satisfaction regarding physical conditions in my work area (noise, lighting, cleanliness, order, etc.)

My degree of satisfaction with the type of training I receive from the company is

I consider that I receive sufficient supplies necessary to carry out my work activities

Degree of satisfaction I feel working with my coworkers

Corresponds to the frequency with which my labor rights are respected in my company

I look for mechanisms to remove the obstacles I identify in achieving my work goals and objectives

Regarding my current hiring contract status, I feel

My degree of satisfaction working at this company (compared to other institutions I know) is

Regarding the functions I perform in this company, my level of satisfaction is

My level of satisfaction with the use I make of my skills and potential in this job is

Regarding the recognition I receive from other people for my work, I feel

My level of satisfaction with my performance as a professional in this job is

Regarding the quality of basic services in my housing, I am

How much do I perceive that my work is useful to other people?

I have full physical, mental, and social capacities to perform my daily activities (dressing, walking, commuting, eating, etc.)

My job allows me access to sufficient quantity and quality of food

My work contributes to the good image that the company has with its users

It is the degree of commitment I feel toward achieving my objectives regarding work

I consider that the personal fulfillments I have reached are due to my work at the company

My capabilities improve by being in this job

I consider that my job has allowed me to provide the necessary care to preserve my physical, mental, and social capacities

My job allows me to fulfill the activities I plan for when I am outside of work hours

My work activities allow me to participate in the care of my family (children, parents, siblings, and/or others)

Section 5: Workplace Violence Questionnaire (IVAPT-PANDO)

Below is a series of situations that may occur in the workplace. Please read each item carefully and select the option that best describes the frequency with which you have experienced each situation in your current job during the past 6 months.

Response Options (Likert Scale)

Never

Occasionally

Sometimes

Frequently

Always

--

Statements:

You are assigned routine, useless, or meaningless tasks

You are assigned tasks well below your capacity or professional qualification

Your work is evaluated unfairly, disproportionately, or with bias

You are given deadlines that are impossible to meet

Information needed to do your job is hidden or withheld from you

You are assigned work involving excessive risk or unsafe conditions

Your presence is ignored, you are given the "silent treatment," or people act as if they don't see you

Your colleagues or superiors refuse to speak to you

You are isolated or physically separated from your coworkers

You are forbidden or restricted from communicating with your coworkers

You receive constant criticism about your personal or private life

People whisper, slander, or spread false rumors about you

Your physical appearance, voice, or gestures are ridiculed or mocked

You are shouted at, insulted, or spoken to using offensive language

You receive verbal threats or intimidating insinuations

Your work is excessively or oppressively monitored and scrutinized

You are assigned a disproportionate or unmanageable workload

Your work achievements are undervalued, dismissed, or downplayed

Errors you did not commit are blamed on you

Your duties or responsibilities are changed without justification
